# The effects of cadmium or zinc multigenerational exposure on metal tolerance of *Spodoptera exigua* (Lepidoptera: Noctuidae)

**DOI:** 10.1007/s11356-013-2409-z

**Published:** 2013-12-20

**Authors:** Alina Kafel, Katarzyna Rozpędek, Elżbieta Szulińska, Agnieszka Zawisza-Raszka, Paweł Migula

**Affiliations:** Department of Animal Physiology and Ecotoxicology, University of Silesia, Bankowa 9, PL 40-007 Katowice, Poland

**Keywords:** Antioxidant defence, Multigenerational stress, Heavy metal exposure

## Abstract

The effects of ten generational zinc or cadmium pre-exposure on metal tolerance among beet armyworm *Spodoptera exigua* individuals were compared. These effects were assessed in animals from the 11th generation, reared on a diet either uncontaminated or contaminated with metal (cadmium or zinc). The survival rate of larvae and the degree of metal accumulation (in larvae, pupae and moths; among larval organs: gut and fat body) were analysed. Catalase, superoxide dismutase and glutathione transferase activity in larval organs of individuals subjected to different metal treatments were also measured. Animals transferred from control rearing to metals (cadmium or zinc) in the 11th generation, as well as those from multigenerational zinc treatment, but not from multigenerational cadmium treatment, had a significantly lower survival rate than control animals. Insects from the groups with the high metal treatment had high bioaccumulation factors (above 3.7 and 2.3 following cadmium and zinc, respectively). Cadmium (but not zinc) pre-exposure had a significant effect on metal accumulation in larvae. Multigenerational metal pre-exposure seemed to have mainly a negative effect on glutathione transferase activity in the gut of larvae from the 11th generation, in the case of the individuals exposed to metal other than that used in pre-exposure treatment or kept in control conditions. However, in the case of zinc pre-exposure, such effect was only apparent when zinc was replaced by cadmium. The long-term effect of cadmium on catalase activity in larvae was found.

**Electronic supplementary material:**

The online version of this article (doi:10.1007/s11356-013-2409-z) contains supplementary material, which is available to authorized users.

## Introduction

The toxic effects of metals (related to industrial and urban contamination) on insects have been extensively studied for many years. Zinc (Zn) and cadmium (Cd) have been especially examined due to many aspects of their pro-oxidative nature (Bertin and Averbeck 2006; Gallego et al. 2007). Zinc is an essential metal, associated with about 300 enzymes which perform multiple actions in the organism, including regulation of cellular processes, antioxidant response and protection against apoptosis. Notwithstanding, in excess, it may severely disturb the cellular environment, increasing oxidative stress (Coleman 1992; Koh 2001). Cadmium is not an essential trace metal. Its toxicity to animals depends on their developed tolerance mechanisms. The detrimental action of cadmium takes several forms, such as blocking of signalling receptors, interactions with kinases or phosphatases, induction of oxidative stress, or genotoxic and necrotic effects. Recently, such actions were summarised in the review of Zervas et al. (2010).

In insects, various mechanisms counteract the toxic effects of metals. They are necessary for organisms inhabiting in metalliferous areas or living under industrial stress. Among these mechanisms, that are important for reduction of metal toxicity, are those related with the ability to form and excrete intercellular granular concretions in which metals are stored (van Straalen and Roelofs 2005). Pathways for metal elimination, including binding with metallothioneins, and enzymatic and non-enzymatic antioxidative processes, are another important element of organismal protection against toxic metal action (Korsloot et al. 2004; Boyd 2007; Roelofs et al. 2007; Augustyniak et al. 2009). Oxidative stress caused by metals needs effective antioxidant defence systems to ameliorate potential subsequent tissue damage. Among various ways of antioxidant defence against oxidative stress, catalase (CAT), superoxide dismutase (SOD) and glutathione transferase (GST) play a crucial role. SOD, together with CAT, is of key importance in alleviating damage caused by free radicals. Many studies have proven the validity of these enzymes as biomarkers of general oxidative stress in metal-contaminated areas (e.g. Augustyniak and Migula 2000; Migula et al. 2004; Augustyniak et al. 2009). Metal stress may up-regulate genes of antioxidant enzymes, as was demonstrated in the case of glutathione transferase of *Drosophila* individuals under Zn or Cd stress. This phenomenon was observed 6 h after transfer of animals to metal-supplemented diet. And it was suggested that such response of GST may be important for the reduction of lipid peroxidation products after heavy metal exposure (Yepiskoposyan et al. 2006). In the maintaining of the homeostasis, the importance of the increase of SOD expression and activity in the Cd-stressed clam *Mactra vereniformis* were shown by Fang et al. (2010).

Either the alimentary tract or fat body is important target of toxic metals. It is emphasised that fat body is involved in multiple homeostatic functions, regulating nutrient synthesis and storage, ontogenetic development or providing several metabolic pathways (Keeley 1985; Kafel et al. 2003; Xia et al. 2005). The alimentary tract plays an important role as a major organ of heavy metal storage, from which metals may be excreted during gut epithelium renewing. Such a phenomenon was registered in springtails *Orchesella cincta* reared in laboratory conditions on algae contaminated with cadmium (Hensbergen et al. 2000). Metal excretion through this pathway may be related to metal tolerance of insects from heavy metal-polluted sites (van Straalen and Roelofs 2005).

There are several examples of increased metal tolerance of animals, which were collected from polluted environments or reared in their F1 generations in metal-stressed laboratory conditions. For example, field-selected tolerance to heavy metals of the springtail *O. cincta* (Colembolla) was shown by Roelofs et al. (2007). The majority of examples (usually recognised as better tolerance to a specific metal) were categorised as physiological adaptations, but not genetic adaptations (Janssen et al. 2009).

Increased resistance to one metal may change biological responses to the other. Postma et al. (1995) indicated higher sensitivity to excessive Zn exposure in a cadmium-tolerant *Chironomus riparius* strain than in cadmium-intolerant (control) populations. Such interactions (also found with other chemical or natural stressors) have been demonstrated for crickets and ants (Migula et al. 1989, 1997).

Some herbivorous insects efficiently utilise various parts of metal-hyperaccumulating plants as they can accumulate and sequester large amounts of metals within their body (Boyd 2007; Migula et al. 2011). Herbivore polyphagic species can overcome the toxic effects of metals merely by avoiding certain foods (Behmer et al. 2005). Under laboratory conditions, examination provided on beet armyworm *Spodoptera exigua* larvae from cadmium strains (reared over 30 generations on larval diet contaminated with metal) revealed a tolerance development to cadmium exposure, when comparing them with larvae from control strain. In this study, the importance of antioxidant processes in larval haemolymph was emphasised (Kafel et al. 2012b).

In the present study, we tested whether the tolerance of *S. exigua* to dietary Cd or Zn after constant metal exposure lasting ten consecutive generations would increase. This study aimed to test the hypothesis that persistently acting pro-oxidants, such as heavy metals, would enhance constitutive levels of antioxidant defence over multiple generations. Animals were divided into separate rearing strains: cadmium, zinc and control. The effects of multigenerational metal rearing were measured in larvae from the 11th generation. Larvae from this generation from each examined strain were divided into groups fed on a diet contaminated either with zinc or cadmium, or on an uncontaminated diet. We assumed that divergent selection to metal stress through ten generations of *S. exigua* would evolve traits supporting improved tolerance to Zn or Cd. Selective changes were expected in the constitutive antioxidant defence of larvae under pressure of Zn or Cd.

## Material and methods

### Insects

The beet armyworm *S. exigua* Hübner (Lepidoptera: Noctuidae) used in our experiments is a polyphagous insect, recognised as a serious pest affecting many vegetable crops, and is widely distributed across Mediterranean Europe, Asia, America, Africa and Australia (Goh et al. 1991; Greenberg et al. 2001).

This experiment was performed on randomly chosen insects from a laboratory stock, bred for many generations on a semisynthetic diet (Poitout and Bues 1974). Larvae were kept on a diet containing a mixture of wheat germ, yeast powder, casein, sucrose, Wesson salt mixture, Vanderzant vitamin mixture, sulphate streptomycin, formaldehyde, methyl *p*-hydroxybenzoate, agar and water. Food was given ad libitum. A temperature of laboratory breeding (25 ± 1 °C) and a light/dark cycle of 16:8 h were maintained. Larvae and pupae were kept in 90 mm in diameter Petri dishes (about ten individuals in each). Moths were kept in plastic cages (volume 2.5 L) with access to a water solution of honey (10 % *v*/*v*) as food.

### Experimental design

**Fig. 1:**
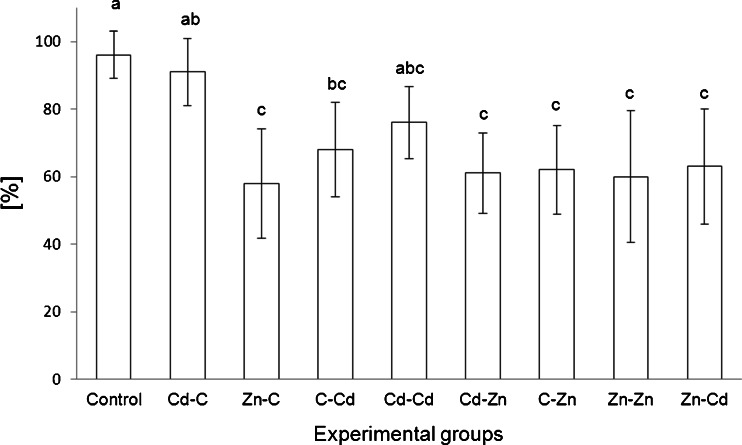
Survival (in percentage) of *S. exigua* larvae from three strains (with control, cadmium or zinc pretreatment through ten generations) divided into experimental groups accordingly to exposure to control diet or contaminated by cadmium or zinc. *Symbols* stand for the experimental groups according to metal exposure of 11th generation larvae to control diet: control (originated from control strain), Cd–C (originated from cadmium strain), Zn–C (originated from zinc strain); cadmium-contaminated diet: C–Cd (originated from control strain), Cd–Cd (originated from cadmium strain), Zn–Cd (originated from zinc strain); zinc: C–Zn (originated from control strain), Cd–Zn (originated from cadmium strain), Zn–Zn (originated from zinc strain). Mean value ± SD. *Different letters* denote significant difference among experimental groups (Kruskal–Wallis test, *P* < 0.05)

The larvae of *S. exigua* were given standard diet enriched with either 44 mg Cd or 200 μg Zn/g dry weight (as chlorides) through ten consecutive generations. Nominal Cd concentrations in the diet were in accordance with the measured values. The procedure of measurement of metal concentration is presented in the next subsection. The average cadmium concentration in the control diet was 1.03 ± 0.21 μg/g dry weight. In the cadmium-spiked diet, it was 41.3 ± 8.3 μg/g dry weight or the equivalent of 93.8 % of the Cd level in a nominal diet (*P* < 0.05). The concentration of Zn in the control diet was 50.3 ± 7.4 μg/g dry weight and in the Zn-spiked diet, 203.0 ± 18.2 μg/g dry weight. When baseline and supplemented concentrations of Zn (50.3 + 200 μg/g dry weight) were added, the measured concentration in the metal-contaminated diet equalled 81.2 % of the total expected Zn level. The cadmium and zinc concentrations used in this study are representative of those found in some contaminated or metalliferous environments (Xian 1989; Wang et al. 2009) and were a result of earlier studies to obtain a mild level of mortality of larvae, about 25–35 % in comparison with control animals (this happened each time when animals from the control strain were exposed to metal; see Table 1 and Figs. 1 and 2 in the Electronic Supplementary Material). Three experimental stock groups (strains) of insects were established: the control strain (larvae fed on the control diet), the cadmium strain (larvae fed on the cadmium-contaminated diet) and the zinc strain (larvae fed on the zinc-contaminated diet). Freshly hatched caterpillars of the 11th generation from each strain were then divided into experimental groups (which differed in having been reared on the control diet, the cadmium- or the zinc-contaminated diet). The experimental group names were created using two abbreviations accordingly to rearing through ten generations—the first abbreviation, and in the 11th generations—the second abbreviation (the used abbreviations: C—control rearing; Cd and Zn—rearing larvae on the cadmium or zinc diet, respectively). In total, animals from nine experimental groups were examined. In each experimental group, 100 individuals were reared for observation of larval survival rate, and 80 individuals were reared for metal concentration and enzyme activity determinations.

Metals were given throughout the period of larval development. The number of individuals which survived from first larval instar to pupation was recorded.

### Metal analysis in *S. exigua* stages and in larval food

Concentrations of Zn and Cd in the body were determined for 3-day-old last-instar larvae (fifth instar) and also for 3-day-old pupae and moths, using a Solaar Unicam 939 (Unicam Limited, Cambridge, United Kingdom)  atomic absorption spectrometer, as described by Kafel et al. (2012a). A single individual (of larvae, pupae or imagoes) constituted one sample. Insect as well as food samples (about 50 mg of dry weight each) were digested in Suprapur Nitric Acid (Merck, Darmstadt, Germany). The digests were used for quantitative Cd and Zn determination using an air–acetylene flame for Zn and a PU-93 090X graphite furnace for cadmium. Methodological accuracy was confirmed by analysis of certified material: bovine liver SRM-1577b (Department of Commerce, National Institute of Standards and Technology, Gaithersburg, MD, USA). The bioaccumulation factor (BAF), corresponding to the ratio of the Cd concentration in the larvae (in micrograms per gram of dry mass) over the contamination of the larval diet (in micrograms per gram of dry mass), was calculated for each experimental group. For metal measurements, whole individuals and those used for tissue dissection (each time, tissue was collected from three larvae to make one sample) were randomly chosen from each experimental group.

### Enzyme assays

Enzyme activities, including catalase, Cu–Zn–superoxide dismutase and glutathione S-transferase, were measured in the gut and the fat body isolated from actively feeding fifth-instar larvae. Three individuals were pooled as one sample. Larvae, anaesthetized on ice, were dissected in KCl solution (11.5 mg/ml) under a stereomicroscope. Each isolated organ was washed three times in the same solution, then gently homogenised in 5 mM Tris–HCl buffer, at pH 7.4, containing sucrose (200 g/L), 1 mM ethylenediamine tetraacetic acid (EDTA), 1 mM phenylmethylsulfonyl fluoride and 1 mM dithiothreitol. Homogenates were centrifuged for 10 min at 1,000 *g* at 4 °C, and the obtained supernatants were centrifuged for 15 min at 15,000 *g* at 4 °C. The final supernatants were kept at −70 °C until analysis in enzymatic assays.

CAT activity was measured at 240 nm in a mixture containing 0.05 M phosphate buffer (pH 7.0), sample and 10 mM H_2_O_2_ according to the method of Aebi (1984). In the blank sample, phosphate buffer replaced H_2_O_2_. Readings of 30 s within a linear range of reaction rate were carried out on a UV/Vis Cecil 3000 Spectrophotometer (Cecil Instruments Limited, Cambridge, England) using two chambers loaded simultaneously—one with a blank sample and the other with an experimental sample. Enzyme activity was expressed in nanomoles of H_2_O_2_ per minute per milligram of protein.

SOD assay was performed as described by Misra and Fridovich (1972) based on spontaneous auto-oxidation of epinephrine to adrenochrome. Enzyme activity was measured in two steps in a mixture of 0.05 M Na_2_CO_3_ + NaHCO_3_ buffer, pH 10.2, with 0.1 M HCl, at pH 2.0, also containing epinephrine (7 mg in 4 ml 0.1 M HCl). In the first step, the rate of epinephrine auto-oxidation was established at 480 nm, using the Cecil UV/Vis 3000 spectrophotometer. The absorbance rate for 0.33 mM of epinephrine solution without SOD is 0.025 absorbance units per minute. In the second step, following the dilution of the sample, a 50 % inhibition of epinephrine auto-oxidation to adrenochrome was established. The accuracy of the assay was validated with a commercial SOD standard from bovine erythrocytes (Sigma, S-2515). The activity unit of Misra and Fridovich (1972) is defined as the amount of enzyme that inhibits 50 % of the control reaction of epinephrine auto-oxidation per minute per milligram of protein.

GST activity was measured as that by Yu (1982) using 1-chloro-2,4-dinitrobenzene (CDNB), which conjugates with glutathione as the substrate at 340 nm. The reaction medium contained 90 μl of 1 mM of reduced glutathione in 0.05 M of phosphate buffer, at pH 7.4, and 9 μl of sample. The reaction began after the addition of 1 μl of 15 mM CDNB in 96 % ethanol. Changes of absorbance were recorded, within the linear range of reaction rate, over a 3-min period. Blank values (without samples) were subtracted to yield the final absorbance values. Enzyme activity was expressed as nanomoles of glutathione conjugates per minute per milligram of protein.

Protein content was measured according to Bradford (1976), using bovine serum albumin as a standard.

### Statistical analysis

Data were presented as the mean values ± standard deviation. Where required, the data were log-transformed and then checked for homogeneity and normality. The effects of variance components and possible interactions were calculated using one- and two-way analysis of variance. For survival and larval body weight analysis, a non-parametric Kruskal–Wallis test was applied. Where the *F* estimate exceeded a probability of 0.05, the differences were considered significant. Multiple regressions were used to verify which variables had a significant effect on measured endpoints (enzymatic activity). The independent variables were insect strains exposed for ten generations to Zn or Cd, body burdens of both metals in the 11th generation and target organs (the gut and the fat body) (Statistica package, version 8.0 for PC).

## Results

### *S. exigua* performance in the 11th generation

Development of the control strain of *S. exigua* larvae kept on the standard diet was successful: nearly 95 % of individuals survived the period from hatching to the early phase of pupation. Exposure to cadmium led to a decrease in the survival rate among individuals originating from the control (by about 30 %, *P* < 0.05) and zinc strains (by about 40 %, *P* < 0.05), but not from the cadmium strain (the mean value of survival rate was equal to 76 % and was similar to earlier mentioned groups: control, C–Cd and C–Zn). Exposure to zinc caused a significant decrease (by about 40 %) in the rate of larvae survival independently of origin (*P* < 0.05) (Fig. 1).

### Metal accumulation

Metal concentrations were measured in the 3-day-old fifth-instar larvae, pupae and imagoes. Generally, treatment with metal (cadmium or zinc) in the 11th generation led to significant increases of metal concentrations in all examined stages when compared with the data from the control group (Table 1).

**Table 1:** Concentration of Cd and Zn in larvae, pupae and moths on the third day of each developmental stage of *S. exigua*

	Cd concentration [mg/kg dry mass]			Zn concentration [mg/kg dry mass]		
	Larvae (L5) (*N* = 10)	Pupae (*N* = 9)	Moths (*N* = 7)	Larvae (L5) (*N* = 10)	Pupae (*N* = 9)	Moths (*N* = 7)
Control treatment in the 11th generation						
Control	3.4 ± 2.1a^#^	1.5 ± 1.7a^#^	1.9 ± 2.0a^#^	118 ± 8a^#^	71 ± 7a^§^	135 ± 40a^#^
Cd–C	2.9 ± 2.5a^#^	1.3 ± 2.0a^#^	0.2 ± 0.4a^#^	119 ± 12a^#^	59 ± 11a^§^	137 ± 40a^#^
Zn–C	3.7 ± 3.6a^#^	1.4 ± 1.5a^#^	0.6 ± 0.4a^#^	115 ± 38a^#^	72 ± 26a^§^	142 ± 28a^#^
Cadmium treatment in the 11th generation						
C–Cd	155.3 ± 29.8b^#§^	174.9 ± 15.4b^#^	132.4 ± 36.1c^§^	149 ± 23a^#§^	130 ± 14b^#^	179 ± 41a^§^
Cd–Cd	162.9 ± 44.8b^#^	140.6 ± 25.7b^#^	87.4 ± 17.3b^§^	95 ± 40a^#^	117 ± 32b^#^	179 ± 24a^§^
Zn–Cd	178.6 ± 16.7b^#^	146.1 ± 19.2b^#§^	127.2 ± 40.7c^§^	154 ± 8a^#^	132 ± 23b^#^	168 ± 52a^#^
Zinc treatment in the 11th generation						
C–Zn	3.2 ± 2.2a^#^	0.7 ± 1.0a ^#^	3.1 ± 3.3a^#^	773 ± 122c^#^	361 ± 95c^§^	715 ± 69b^#^
Zn–Zn	3.1 ± 1.7a^#^	1.2 ± 1.7a^#^	1.5 ± 0.9a^#^	667 ± 143bc^#^	329 ± 53c^§^	1,014 ± 335c&
Cd–Zn	6.9 ± 3.8a^#^	1.2 ± 0.8a^§^	3.9 ± 5.2a^#§^	453 ± 53b^#^	402 ± 54c^#^	601 ± 102b^§^

Mean value ± SD. Different letters indicate significant differences among the experimental groups within a developmental stage (in columns). Different symbols (#, §, &) indicate significant treatment-dependent differences among developmental stages (in rows) (ANOVA, Tukey test, *P* < 0.05). Descriptions of the strains and experimental groups are given in Fig. 1

In cadmium-treated larvae from different strains, similar concentrations of metal were recorded. In zinc-treated larvae originating from the cadmium strain (Cd–Zn group) significantly lower Zn concentrations were noted in comparison to those of larvae originating from the control strain (C–Zn group). The BAF for cadmium calculated for the larvae exposed to this metal was 3.8 for C–Cd group and 4.3 for Zn–Cd group. The BAF for Zn ranged from 2.2 for Cd–Zn group to 3.8 for C–Zn group. Pupae from particular metal-stressed groups (either in the case of cadmium or zinc treatment) had similarly higher level of this metal over control levels. Some variations in metal accumulation were found between moths from different metal strains. We detected significantly lower Cd concentrations in moths originated from the cadmium strain (Cd–Cd group) compared with animals from the control strain (C–Cd group) and the zinc strain (Zn–Cd group). On the contrary, a higher Zn concentration was detected in moths originated from the zinc strain (Zn–Zn group) compared to those from control strain (C–Zn group) and from the cadmium strain (Cd–Zn group) (Table 1).

When the metal concentrations among subsequent developmental stages were compared, the lowest Cd content was measured in moths from the groups treated with this metal in the 11th generation (groups: C–Cd, Cd–Cd and Zn–Cd). Among analysed developmental stages, the lowest Zn content was detected in pupae from experimental groups treated with zinc. Moths from the 11th generation from groups exposed to Zn had higher Zn concentrations than those observed in larvae and pupae (Zn–Zn and Cd–Zn groups) (Table 1).

Metal-stressed larvae accumulated much higher amounts of metal in their gut and fat body than larvae from the control group. Metal concentrations in the gut of larvae treated with cadmium or zinc were similar independently on their strain origination (control, cadmium or zinc) (Table 2).

**Table 2:** Concentration of Cd and Zn in the gut and fat body of *S. exigua* in the last instar of larvae

Group	Cd concentration [mg/kg dry mass]		Zn concentration [mg/kg dry mass]	
	Gut	Fat body	Gut	Fat body
Control treatment in the 11th generation				
Control	0.2 ± 0.1a^#^	0.5 ± 0.6a^#^	268 ± 49a^#^	72 ± 21a^§^
Cd–C	1.0 ± 0.9a^#^	1.0 ± 1.5a^#^	80 ± 30a^#^	58 ± 7a^#^
Zn–C	0.7 ± 0.5a^#^	0.4 ± 0.2a^#^	115 ± 38ab^#^	72 ± 26a^#^
Cadmium treatment in the 11th generation				
C–Cd	225.5 ± 92.4b^#^	34.0 ± 10.5c^§^	195 ± 60ab^#^	78 ± 6a^§^
Cd–Cd	422.2 ± 145.4b^#^	34.5 ± 9.8c^§^	172 ± 48a^#^	78 ± 28a^§^
Zn–Cd	371.2 ± 93.2b^#^	9.3 ± 4.6b^§^	153 ± 8b^#^	58 ± 20a^§^
Zinc treatment in the 11th generation				
C–Zn	0.7 ± 0.2a^#^	2.4 ± 2.2a^#^	1,081 ± 241c^#^	85 ± 28a^§^
Zn–Zn	1.7 ± 2.6a^#^	1.2 ± 1.7a^#^	666 ± 143c^#^	329 ± 53a^§^
Cd–Zn	5.0 ± 4.4a^#^	0.3 ± 0.2a^#^	1,405 ± 633c^#^	68 ± 20a^§^

*N* = 5, mean value ± SD. Different letters indicate differences among experimental groups in the same organ (in columns), and the symbols # and § indicate differences between organs in the same treatment (in rows) (LSD test, *P* < 0.05). Descriptions of the strains and experimental groups are given in Fig. 1

In the larval fat body, opposite results of metal accumulation were observed. Lower concentrations of cadmium were measured in the fat body of larvae originated from the zinc strain (Zn–Cd group) than in the fat body of other strains larvae exposed to cadmium (groups Cd–Cd and Zn–Cd). Among all examined groups, the highest Zn concentrations were measured in the fat body of larvae from Zn–Zn group. Moreover, Cd concentrations in the gut of metal-stressed larvae were more than 6, 12 and 39 times higher than in their fat body (larvae from C–Cd, Cd–Cd and Zn–Cd groups, respectively). Zn concentrations were also much higher in the gut than in the fat body: in the case of larvae from Zn–Zn groups, concentrations of this metal were about two times higher, while in the case of zinc-stressed larvae from other strains (C–Zn and Cd–Zn groups), these differences were more evident (Table 2).

### Antioxidant response

The cellular endpoints were the activities of three enzymes: CAT, SOD and GST in the gut and the fat body of the fifth stage of *S. exigua* larvae. Both organs play an important role in detoxification and were selected in this study for assays of antioxidative enzymes.

Generally, CAT activity was higher in the fat body than in the gut of fifth-stage larvae. Individuals from Cd–Cd group had higher CAT activity in both organs than those from C–Cd group. The highest activity of CAT was measured in the fat body of larvae of Zn–Cd group. After zinc treatment, larvae (originated from different strains) differed in CAT activity measured in their fat body, but not in their gut. CAT activity in the fat body of larvae from Cd–Zn group was about three times higher than that measured in larvae from C–Zn and Zn–Zn groups. For larvae derived from the zinc strain and transferred to cadmium-contaminated diet (comparison of larvae from Zn–Cd and C–Cd groups), a significant, more than threefold increase of the enzyme activity in the fat body was found (Table 3).

**Table 3:** Activity of the antioxidant enzymes in the gut and fat body in the last instar of *S. exigua* larvae

	CAT [nmol/min/mg protein]		SOD [units/min/mg protein]		GST [nmol/min/mg protein]	
	Gut	Fat body	Gut	Fat body	Gut	Fat body
Control treatment in the 11th generation						
Control	142 ± 66ab^#^	457 ± 202b^§^	2.9 ± 0.3a^#^	3.1 ± 0.7a^#^	74 ± 19a^#^	69 ± 22bc^#^
Cd–C	196 ± 82b^#^	1,038 ± 164d^§^	6.8 ± 2.3b^#^	6.4 ± 2.6b^#^	42 ± 23b^#^	45 ± 16cd^#^
Zn–C	89 ± 23a^#^	706 ± 170c^§^	4.1 ± 2.5ab^#^	3.6 ± 1.5a^#^	40 ± 9bc^#^	29 ± 7cd^#^
Cadmium treatment in the 11th generation						
C–Cd	176 ± 58b^#^	417 ± 50b^§^	3.1 ± 1.2a^#^	4.4 ± 1.2a^#^	59 ± 31ab^#^	102 ± 32a^§^
Cd–Cd	477 ± 54c^#^	954 ± 107cd^§^	3.7 ± 0.5a^#^	2.5 ± 0.8a^#^	71 ± 8ab^#^	51 ± 4bc^§^
Zn–Cd	150 ± 56ab^#^	1,385 ± 142e^§^	5.9 ± 2.2b^#^	3.0 ± 1.7ab^§^	19 ± 6c^#^	41 ± 20cd^§^
Zinc treatment in the 11th generation						
C–Zn	121 ± 52a^#^	236 ± 79a^#^	4.0 ± 0.7a^#^	4.1 ± 1.0ª^#^	69 ± 9ab^#^	72 ± 13b^#^
Zn–Zn	113 ± 37a^#^	322 ± 153ab^§^	3.8 ± 1.1a^#^	3.8 ± 1.7a^#^	66 ± 12ab^#^	52 ±10bc^#^
Cd–Zn	102 ± 46a^#^	816 ± 193c^§^	3.2 ± 1.0a^#^	3.6 ± 1.2a^#^	33 ± 14c^#^	28 ± 14d^#^

*N* = 5, mean value ± SD. Different letters indicate significant differences among experimental groups in the same organ (in columns); symbols # and § indicate significant differences between organs in the same treatment (in rows) (LSD test, *P* < 0.05). Descriptions of the strains and experimental groups are given in Fig. 1

Cadmium-stressed larvae from the control strain or from cadmium strain were characterised by similar SOD activities as measured in the control larvae. Similarly, zinc treatment did not cause any effect on the SOD activity, irrespectively of larvae origination (from control, cadmium or zinc strain). When larvae from Cd strain were transferred to a metal-free diet, their SOD activity in the gut reached a higher, over twofold level than that in larvae from the control strain (comparison of Cd–C and control groups). In turn, when animals from the Zn strain were exposed to the cadmium-contaminated diet, SOD activity in their gut was almost twofold higher than that in similarly treated larvae from the control strain (Zn–Cd vs C–Cd group). Additionally, only in the case of larvae from Zn–Cd group that the activity of the enzyme differed between the gut and fat body (lower in the latter) (Table 3).

Generally, in animals transferred from one metal-treated strain to the other metal treatment or to control conditions, a decrease in GST activity in the gut of larvae was detected. Such a trend was recorded in both organs of larvae from Cd–Zn group. In larvae from control strain exposed to cadmium (group C–Cd), almost two times higher GST activity was registered than in larvae from control and Cd–Cd group. The activity of GST was higher in the gut than in the fat body of larvae from control and Cd–Zn groups (Table 3).

In order to compare the enzyme activity to metal exposure models, we calculated two main effects, with Cd and Zn concentrations as covariates. It appeared that a covariate (Cd concentration in organs) had an effect only on SOD activity. Thus, for GST and CAT activities, we applied two-variance (ANOVA) data calculation, while for SOD, we applied ANCOVA. Exposure to metal was a substantial factor that influenced all examined enzyme activity. CAT activity was also dependent on the type of organ (Table 4).

**Table 4:** Summary of multi-factor analysis of variance for enzymes (SOD, GST and CAT) activity in the gut and fat body of the instar of larvae of *S. exigua*. Only in the case of SOD activity analysis was the covariate concentration of Cd in organs significant (*P* < 0.05)

	*df*	SS	MS	*F*	*P*
		SOD			
Covariates					
Cd in organs	1	8	8	4	0.046
Main effects					
Metal treatment	8	59	7	4	0.001
Organ	1	NS	NS	NS	
Group × organ	8	33	4	2	0.049
		GST			
Metal treatment	8	29,908	3739	13	0.00001
Organ	1	NS	NS	NS	
Group × organ	8	7,838	980	3	0.002
		CAT			
Metal treatment	8	3,748,322	468,540	38	0.00001
Organ	1	6,306,864	6,306,864	505	0.00001
Group × organ	8	2,610,622	326,328	26	0.00001

*NS* not significant

Multiple regressions were used to verify which variables affected the above-mentioned endpoints in relation to independent values (intoxication with metals for ten generations, intoxication with metals in the 11th generation and target organ). These produced different results for each enzyme. The regression model explained 73 % of total variability in CAT activity (*r* ^2^ = 0.752; *R* ^2^ adjusted = 0.727; *P* < 0.00001) in terms of the significant effect of Cd added to the diet of larvae of subsequent generations (*P* < 0.00001). Zn exposure in the 11th generation (*P* = 0.18) and Zn concentration in target organs were insignificant for CAT variability (*P* = 0.43).

The model for catalase activity is as follows (Eq. 1):

1 $$ {Y}_{\left(\mathrm{CAT}\right)}=8.8\times {\mathrm{Cd}}_{10\mathrm{gen}}+1.4\times {\mathrm{Zn}}_{10\mathrm{gen}}+15.9\times {\mathrm{Cd}}_{11\mathrm{gen}}+400.1\times \mathrm{org}-84.9\times {\mathrm{org}}_{\mathrm{Cd}}-167 $$

where

*Y*_(CAT)_: activity of catalase
Cd_10gen_: Cd intoxication of *S. exigua* larvae for ten generations
Zn_10gen_: Zn intoxication of *S. exigua* larvae for ten generations
Cd_11gen_: Cd intoxication of *S. exigua* larvae in the 11th generation
Org: target organ (the gut or the fat body)
org _Cd_: Cd concentration in organs of *S. exigua* larvae.

A similar model failed to explain the variability in SOD activity (*r* ^2^ = 0.165; *R* ^2^ adjusted = 0.08; *P* < 0.07).

For GST activity, the model explained only 29 % of total variability (*r* ^2^ = 0.354; *R* ^2^ adjusted = 0.29; *P* < 0.00001) on the basis of two significant independent parameters: exposure of two *S. exigua* strains to Cd (*P* < 0.000) or Zn (*P* < 0.000) through ten generations.

## Discussion

### *S. exigua* survival

A negative impact of metals (Cd or Zn) on the survival of *S. exigua* larvae was observed in our study (comparison of C–Cd and C–Zn with control group; Fig. 1). Similar negative effects of metals were obtained in other studies on herbivorous insects, in which Cd and Zn were used as stress factors. A significant effect of zinc, added to larval diet, on larval mortality, pupation and adult emergence was demonstrated for *Heliothis virescens* by Popham and Shelby (2006). Noret et al. (2007) demonstrated such effects in *Issoria lathonia* while studying on the development of this nymphalid butterfly in Zn-accumulating and non-accumulating *Viola* species. Malacar et al. (2009) indicated the negative effects of cadmium on growth and survival of the grasshopper *Oxya fuscovittata*. Cadmium affected the performance of *Lymantria dispar* larvae, decreasing the body mass of the third and fourth instars (when compared with control animals) (Mirčić et al. 2013). Our observations indicate that Cd and Zn in applied concentrations affected body weight. We noted a low variability in body weight among the larvae from different experimental groups. But the third-stage larvae from the control strain when exposed to metal (cadmium or zinc) had lower body weight than the larvae from the control group (Fig. 3 in the Electronic Supplementary Material).

Lower survival rates of larvae originating from the control strain that were exposed to cadmium (group C–Cd) than in the controls were noted. Larvae originating from the cadmium strain (Cd–C and Cd–Cd) had comparable survival rates to those from control group and also to group C–Cd.

The analysis of Zn effects indicated enhanced tolerance of animals originated from zinc strain to Zn as there was no variation in the survival rate between animals from groups exposed to this metal (C–Zn, Zn–Zn) (Fig. 1). Similarly, there were no observations of increased tolerance in populations of midge *C. riparius* (Diptera) from metal-contaminated streams, despite of their high sensitivity to zinc contamination (Postma et al. 1995). Grześ (2010) examined Zn pollution effects on mortality of ants *Myrmica rubra* and did not find dependence on a metal pollution gradient. It turn, Muyssen et al. (2002) presented significant higher zinc tolerance of *Daphnia magna* populations collected from zinc-contaminated sites.

Animals from the cadmium strain exposed to zinc (Cd–Zn group) had a lower survival rate similar to those from the zinc strain. However, in other studies, examples of consequences on further response to other metal contamination were found (Xie and Klerks 2003). The evidence of higher resistance to copper in zinc-selected *Eisenia fetida* population when compared with control population was presented by Spurgeon and Hopkin (2000).

### Metal accumulation

At the metal concentrations used, *S. exigua* larvae appeared to be macro-concentrators of both metals, taking into account the general classification of metal bioaccumulation rates proposed by Dallinger (1993). In this aspect, *S. exigua* is within the range of other lepidopteran species, for which metal body burdens were reported at levels at least two times higher than in the eaten food. This is not a general rule, as *Parnassius apollo*, feeding on *Sedum telephium* contaminated with Cd or Zn, was identified as an effective deconcentrator of these metals, with metal concentrations in the body lower than that in the diet (Nieminen et al. 2000). The bioaccumulation level of a given metal depends on multiple toxicokinetic factors, including ingestion, distribution, metabolism in a target organ and possible pathways of decontamination (Augustyniak and Migula 2000).

We expected that earlier multi-generation pre-exposure of animals to one of the metals might affect bioaccumulation of the other metal from the diet. In this study, *S. exigua* larvae originated from the cadmium strain and exposed to Zn had a bioaccumulation factor of Zn of at least 1.5 times lower (for Cd–Zn group: BAF_Zn_ = 2.2) than animals from the control strain (C–Zn group) or the zinc strain (Zn–Zn group). In turn, the larvae from the zinc strain, when fed on a Cd-spiked diet, had the highest Cd bioaccumulation factor among Cd-treated groups: Zn–Cd, Cd–Cd and C–Cd. For the remaining two groups, this factor was only about 1.1 times lower than for Zn–Cd group (for the latter group, BAF_Cd_ = 4.3). Among the earthworms *Eisenia*, pre-exposure to Zn had no effect on Cd bioaccumulation, while pre-exposure to Cd affected Zn distribution (Li et al. 2007). The examination of caddisfly *H. californica* also showed no difference in metal accumulation between individuals from populations originated from sites polluted or unpolluted by heavy metals. But it was also emphasised that the difference may be connected with metal compartmentation, which is probably important for the effectiveness of metal detoxification processes (Cain et al. 2006). Similar observations were presented for the earthworm *Dendrobaena octaedra* (Rożen 2006). The presence of detoxification mechanisms such as the aforementioned metallothionein and antioxidant response might be connected with the phenomenon of cross-resistance to metals (Xie and Klerks 2003).

### Metal accumulation during *S. exigua* ontogenesis

For *S. exigua*, the period preceding the moult of the last larval instar to the pupa, at which stage insects cease feeding and reduce mobility, was not effective in cadmium elimination. This was seen in the case of groups C–Cd, Cd–Cd and Zn–Cd (Table 1). Similarly, high cadmium concentrations in pupae and larvae were noted; however, the mean value of Cd concentration was about 14 % higher in larvae than in pupae of Cd–Cd group (Table 1). After the final moult for animals from Cd–Cd group, we noted a significant (over 60 %) reduction of cadmium load in the adult stage. Pupal exuviae were probably the major route for eliminating Cd from *S. exigua* individuals treated with cadmium. Metal binding by the exoskeleton was documented in several species (e.g. Borowska et al. 2004; Robinson et al. 2007). In the case of grasshoppers, *Omocestus viridulus* (Orthoptera) or spiders *Agelena labyrinthica*, Cd elimination via exuvia was negligible (Lindqvist and Block 2009; Babczyńska et al. 2011). The other probable way of Cd elimination from the body of hatching adults was the meconium. Studies on fruit fly *Ceratitis capitata* revealed that approximately 33 % of cadmium was eliminated after eclosion via this route (Kazimirová and Ortel 2000). For individuals from C–Cd group, metal concentration in adult stage was lower by 24 % than in pupal stage; bigger differences between these stages were revealed for individuals from Cd–Cd group—38 % (Table 1). A typical one-compartment, two-phase model (with assimilation and elimination phases) might characterise cadmium toxicokinetics in untreated insects (from the control strain, C–Cd group). In the case of Cd–Cd group, Cd toxicokinetics might be closer to a three-phase model of metal kinetics. The exposure period involves a first short phase of rapid metal accumulation (larvae), followed by possible partial elimination in the second phase (pupae), leading to an equilibrium concentration in the third phase (moth) (Bednarska et al. 2011). This is in agreement with the more general findings of Laskowski et al. (2010), who tested systems of metal elimination over a wide spectrum of terrestrial invertebrates, including insects. The authors examined toxicokinetics of nickel in carabid beetles and earthworms and discussed that with results of other studies on invertebrates exposed to copper or cadmium. They showed immediate accumulation of the metal after exposure and different rates of its elimination in subsequent ontogenetic development phases. Such a model of metal uptake in ontogenesis was not identified in the case of essential metal—Zn, applied in our experiment. This could be due to the multiple roles of zinc as a cofactor of enzymes in metabolism and development of reproductive organs in adults (Coleman 1992). Zn concentration did not change significantly between the larval and pupal stage in Cd–Zn group (Table 1; *P* < 0.001).

The concentration of zinc in pupae was higher than in the larval food: 1.8 times in C–Zn group and 1.7 times in Zn–Zn group (Table 1). Similarly, in pupae of *H. virescens*, Zn concentration was two times higher than that of larval food (Popham and Shelby 2006).

### Metal accumulation in organs

The alimentary tract is the main route of Cd uptake. The gut epithelial cells form the first barrier against toxic substances, including xenobiotic metals. A much lower level of Cd was accumulated in the fat body than in the larval gut (C–Cd, Cd–Cd and Zn–Cd groups). High metal load in the gut epithelial cells has been observed in many insect species under environmental metal stress (Rodrigues et al. 2008). The protective role of the alimentary tract as the first target of metal action is important for metal tolerance due to metal binding with metallothioneins and shedding epithelia cells during the renewing process (van Straalen and Roelofs 2005; Janssen et al. 2009). Studies on *Epilachna nylanderi* (Coccinellidae) feeding on Ni-hyperaccumulating plants showed also rapid replacement of midgut epithelial cells overloaded with nickel by new ones as a significant protective mechanism (Migula et al. 2011). It is questionable whether such mechanisms exist in the case of the cadmium strain of *S. exigua* larvae. The concentration of Cd in the gut of larvae and the ratio of metal concentration in the gut to its concentration in the fat body of larvae was about two times higher for Cd–Cd group when compared with C–Cd group. Despite these differences between the larvae from the Cd–Cd and C–Cd groups, the concentration of metal in their fat body was similar. In a parallel research of cadmium and control strains of *S. exigua*, we revealed that cadmium concentrations in haemolymph of *S. exigua* larvae from cadmium strain were lower than the concentration of the metal in the whole body. Cd concentrations were higher in haemolymph of the cadmium strain larvae than metal-stressed larvae from the control strain. Such situation suggested similar cadmium availability and circulation of Cd to internal organs of larvae, such as fat body (Kafel et al. 2012a). The importance of rapid metal clearance from the internal tissues was suggested e.g. by Leonard et al. (2009). The other mechanism may be related with metal-binding protein management and subcellular distribution of metal (e.g. Ballan-Dufrancais 2002; Long et al. 2010).

### Antioxidant defence

We found a variation in antioxidant defence among metal-stressed larvae, dependent on strain origination. Our study was concerned with the measurements of three enzymes of the antioxidant system in gut and fat body of *S. exigua* larvae. CAT and peroxidases prevent cellular accumulation of H_2_O_2_. Despite organ-dependent differences in CAT activity, a significant increase in the activity of this enzyme was observed in both organs of the larvae from the Cd strain (Cd–Cd group), when compared with data from control and C–Cd groups (Table 3). This increase of CAT activity may play an important role in the development of Cd tolerance in the cadmium strain. A similar effect was shown by Barata et al. (2005), who demonstrated an increase of CAT activity with intensified lipid peroxidation in caddisfly (*Hydropsyche exocellata*) larvae and a positive correlation of CAT activity with Cd body loads. A role for this enzyme in protection against cadmium stress was also demonstrated for nymphs of the orthopteran *Oxya chinensis* (Lijun et al. 2005). Superoxide radicals, generated in the presence of metals, are converted to H_2_O_2_ by SOD. We detected an increase of SOD activity in both target organs of *S. exigua* larvae following cessation of cadmium treatment (Cd–C group) (Table 3). A negative correlation between SOD activity and Cd concentration was also reported for *O. chinensis* (Lijun et al. 2005).

Often, effects observed in the first generation are not necessarily indicative for other generations. The effects may be observed in a non-linear manner in subsequent generations (Salice et al. 2009). Kafel et al. (2003) studied on different effects of Cd (44 and 66 μg/g dry weight of diet) and Zn (200 μg/g dry weight of diet) in two subsequent generations of *S. exigua* on activity of glutathione *S*-transferase. The authors observed enhanced GST activity related to Zn in the fat body in both insect generations, but in Malpighian tubules only in the second generation. The effect of Cd was significant only in the Malpighian tubules, when higher concentrations were used. Effects of metals in the alimentary tract were insignificant. In this study, GST activity was similar in the gut, but different in the fat body of larvae originating from the control and cadmium strains when Cd was applied (comparison of C–Cd with Cd–Cd group). A marked decrease was demonstrated in larval gut after metal replacement (Zn to Cd or Cd to Zn) (Table 3). This suggests that change of stressor pressure may affect functional equilibrium established during earlier generations. Such a reciprocal effect may depend on the degree of direct inhibitory effect of metals on the enzyme, time-dependent regulation of genes of glutathione transferase isozymes and enzymes engaged in glutathione production, or available pool of glutathione reduced for GST action (Yepiskoposyan et al. 2006; Nzengue et al. 2011).

Laboratory studies are helpful in better understanding on metal uptake and its fate in different developmental stages of insects living in field conditions, especially when field studies are carried out along a metal pollution gradient. Migula et al. (2004) studied on the variation in antioxidant enzyme activities in four species of beetles captured from sites along a Zn and Cd pollution gradient. In the case of the herbivorous beetle *Phyllobius betulae* (Curculionidae), GST activity was negatively correlated with metal concentrations in the surrounding environment, but no such correlations were found between Cd or Zn body loads (Migula et al. 2004). In our experiment, the regression model indicated significance for multi-generation exposure to Cd or Zn in only two cases (CAT, GST). Zinc replacement by Cd in two cases (CAT and GST) was the only significant effect in larval gut. Also, Cd replacement by Zn had significance for SOD and GST activity in larval gut. Variables significant for CAT are summarised in Eq. (1) in the “Results” section.

Our analyses conclude that multigenerational exposure of *S. exigua* to metals was probably more successful in the development of tolerance to Cd. The effects of Cd pre-exposure on metal accumulation and CAT activity were observed. Multigenerational zinc pre-exposure did not change larvae tolerance to this metal. Generally, we did not find cross-resistance of cadmium or zinc.

## Electronic supplementary material

Below is the link to the electronic supplementary material.

ESM 1 (DOC 54 kb)

ESM 2 (DOC 116 kb)

ESM 3 (DOC 116 kb)

ESM 4 (DOC 32 kb)
