# Supplementary material for: The effects of cadmium or zinc multigenerational exposure on metal tolerance of Spodoptera exigua (Lepidoptera: Noctuidae)
Source: Environ Sci Pollut Res Int. 2013 Dec 20;21(6):4705–15. doi: 10.1007/s11356-013-2409-z (PMC3945642; doi:10.1007/s11356-013-2409-z)

**Fig. 2.** Survival (in %) of *S.exigua* larvae in subsequent generations exposed to zinc in diet. Explanations: The animals derived from control strain (Control group), zinc strain (Zn-Zn group) and those originated from control strain and in the examined generation exposed to zinc (C-Zn group). Star denotes significant differences with Control group within each generation (Kruskal - Wallis test, P < 0.05).


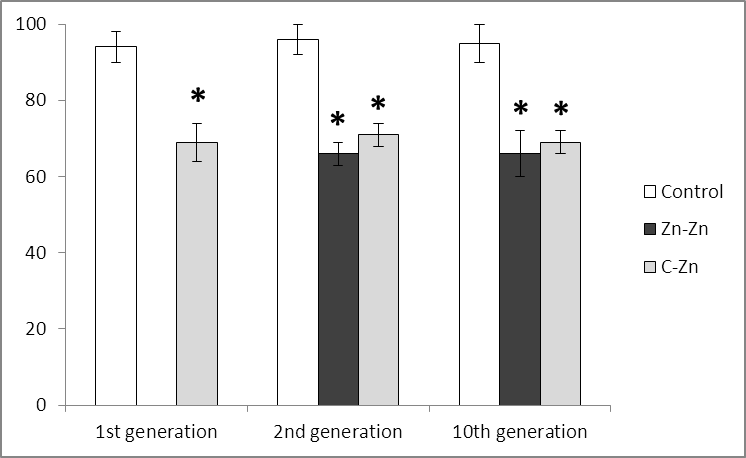

Supplement: Supplementary file 1 — (DOC 54 kb) [file 11356_2013_2409_MOESM1_ESM.doc]
