# Supplementary material for: The effects of cadmium or zinc multigenerational exposure on metal tolerance of Spodoptera exigua (Lepidoptera: Noctuidae)
Source: Environ Sci Pollut Res Int. 2013 Dec 20;21(6):4705–15. doi: 10.1007/s11356-013-2409-z (PMC3945642; doi:10.1007/s11356-013-2409-z)
Supplement: Supplementary file 2 — (DOC 116 kb) [file 11356_2013_2409_MOESM2_ESM.doc]

**Fig. 3.** Wet body weight (in mg) of 3^rd^, 4^th^ and 5^th^ instars (L3 – L5) of *S. exigua* larvae from three strains (control, cadmium or zinc treatment through ten generations) divided into experimental groups accordingly to exposure of the larvae from 11^th^ generation to

(a) control diet - experimental groups dependently on strain origination: control (Control group), cadmium (Cd-C) and zinc (Zn-C),

(b) cadmium - experimental groups dependently on strain origination: control (C-Cd), cadmium (Cd-Cd) and zinc (Zn-Cd),

(c) zinc - experimental groups dependently on strain origination: control (C-Zn), cadmium (Cd-Zn) and zinc (Zn-Zn).

Other explanations: N=10-15, mean ± SD. Star indicates significant differences with the control group (Kruskal -Wallis, P<0.005).
