# Supplementary material for: The effects of cadmium or zinc multigenerational exposure on metal tolerance of Spodoptera exigua (Lepidoptera: Noctuidae)
Source: Environ Sci Pollut Res Int. 2013 Dec 20;21(6):4705–15. doi: 10.1007/s11356-013-2409-z (PMC3945642; doi:10.1007/s11356-013-2409-z)
Supplement: Supplementary file 4 — (DOC 32 kb) [file 11356_2013_2409_MOESM4_ESM.doc]

Table 1. Survival of larvae (calculated together from L1-L5 larval stages) originated from control rearing exposed to:

1. cadmium. (Metal was supplemented to larval diet in concentrations; 22 and 44 µg dry weight of diet in the case of groups: Cd22 and Cd44, respectively),
2. zinc (Metal was supplemented to larval diet in concentrations; 100 and 200 µg dry weight of diet in the case of groups: Zn100 and Zn200, respectively),

Explanations: Different letters denote significant difference among experimental groups (Kruskal – Wallis test, P < 0.05).

a)

| Experimental group | Survival in % (Mean ± SD) | |
| --- | --- | --- |
| Control | 94 ± 4 | a |
| Cd22 | 89 ± 8 | a |
| Cd44 | 74 ± 5 | b |

b)

| Experimental group | Survival in % (Mean ± SD) | |
| --- | --- | --- |
| Control | 94 ± 4 | a |
| Zn 100 | 86 ± 10 | a |
| Zn 200 | 64 ± 4 | b |
